# High Burden and Phenotype-Specific Variability of Pain Polypharmacy in Early Autoimmune Rheumatic Diseases: A 15-Year Real-World Analysis

**DOI:** 10.21203/rs.3.rs-9152435/v1

**Published:** 2026-04-01

**Authors:** Di Lu, Kristen Cunanan, James Cragun, Lauren Vuong, Macarius Donneyong, Michael Weisman, Matthew C. Baker, Anushka Irani, Titilola Falasinnu

**Affiliations:** Stanford University School of Medicine; Stanford University School of Medicine; Stanford University School of Medicine; Stanford University School of Medicine; The Ohio State University; Stanford University School of Medicine; Stanford University School of Medicine; Mayo Clinic; Stanford University School of Medicine

## Abstract

**Background.:**

Pain drives disability and medication use in autoimmune rheumatic diseases (ARDs), even when inflammation is controlled. The extent of pain-related polypharmacy across ARDs and chronic overlapping pain conditions (COPCs) remains poorly characterized. We aimed to quantify first-year pain medication burden following ARD diagnosis and examine variations by disease, COPC status, and time.

**Methods.:**

Using the Merative MarketScan Commercial Claims database, we identified adults newly diagnosed with rheumatoid arthritis, systemic lupus erythematosus, ankylosing spondylitis, psoriatic arthritis, Sjögren’s disease, or systemic sclerosis (2008–2021). We analyzed pain-related medications across ten pharmacologic categories during the first year post-diagnosis. Polypharmacy measures included distinct medication counts, medication categories, ≥ 5 and ≥ 10 medication thresholds, and the Medication Quantification Scale.

**Findings.:**

Among 149,742 adults with newly diagnosed ARDs, 57.6% had at least one COPC. During the first year after diagnosis, patients filled a mean of 9.0 distinct pain medications and 47.9% met criteria for ≥ 5 medications; 30.5% met criteria for ≥ 10 medications. Ankylosing spondylitis demonstrated the highest medication burden (mean 11.6 medications; 57.5% with ≥ 5 medications; 37.9% with ≥ 10 medications). Patients with fibromyalgia had consistently higher medication burden across ARDs and were the only COPC group in whom prescribing increased after 2015. Overall medication burden rose from 2008 to 2014–2015 and declined thereafter; however, absolute levels remained high.

**Interpretation.:**

Pain-related polypharmacy is common early after ARD diagnosis and varies substantially by disease and pain phenotype. Despite modest declines after 2015, medication burden remains high, underscoring the need for phenotype-informed, nonpharmacologic, and deprescribing strategies in rheumatology.

**Funding.:**

NIAMS (K01AR079039).

## INTRODUCTION

Autoimmune rheumatic diseases (ARDs), including rheumatoid arthritis, systemic lupus erythematosus, psoriatic arthritis, ankylosing spondylitis, Sjögren’s disease, and systemic sclerosis, affect more than 1.5 million Americans.^1^ Across these conditions, pain remains a predominant indicator for medication dispensing as well as a direct cause of disability and impaired quality of life, often persisting even when inflammatory activity is well controlled.^2^ This is a central clinical problem: 22–38% of patients with RA experience moderate to severe pain despite adequate inflammatory control, and 60–80% report chronic pain of any degree.^3,4^ In SLE, pain remains one of the most frequently reported symptoms and primary drivers of poor health-related quality of life, even during periods of low disease activity.^5^

Many patients with ARDs have one or more chronic overlapping pain conditions (COPCs), e.g., fibromyalgia, chronic low back pain, migraine, irritable bowel syndrome, and others, which are characterized by nociplastic pain as the unifying mechanism.^6^ These conditions co-occur at high frequency: More than 36–62% of patients with common ARDs carry a concomitant COPC diagnosis, with Sjögren disease patients having the highest prevalence at 62%.^6,7^ Traditionally, fibromyalgia and related COPCs in ARD populations have been viewed as downstream consequences of prolonged, inadequately controlled inflammatory disease.^8,9^ However, accumulating evidence suggests that this paradigm is incomplete. Many patients exhibit predisposing factors (including prior COPC diagnoses and pain vulnerability phenotypes) at or near the time of ARD diagnosis, indicating that these pain conditions are not merely sequelae of persistent inflammation but reflect distinct nociplastic mechanisms involving central sensitization, altered cytokine signaling, and neuroimmune dysregulation.^10,11^ As such, management must extend beyond immunomodulatory therapy and acute pain control. In this context, COPCs are not likely to be the result of persistent ARD exposure but represent distinct pain mechanisms thought to involve central sensitization, altered cytokine signaling, neuroimmune dysregulation, all of which fundamentally require a different approach to pain management, adopting a biopsychosocial perspective and including non-medication based strategies such as education, physical activity and exercise, sleep hygiene and psychological interventions.^12,13^ When COPCs coexist with ARD, pain requires tailored, multimodal treatment beyond acute pain and immunomodulatory-based approaches to management, and clinicians face a challenging treatment problem.^14,15^

Polypharmacy has become standard practice likely in response to the above considerations. Recent real-world data from patients with SLE revealed that patients often cycle through pharmacologic therapies with limited relief.^16^ Among 769 SLE patients, 52.5% received five or more pain-related prescriptions in the first year of treatment, though this declined to 45.3% in the more recent period (2015–2024), suggesting cautious progress toward de-intensification.^16^ However, prescribing patterns remain high and vary substantially by demographic group: 69% of men received five or more pain prescriptions compared with 43% of women.^16^ In recent years, younger patients are increasingly receiving broader ranges of pain medications.^16^ In addition, nearly half of patients initiated on acetaminophen transitioned to therapies such as opioids or glucocorticoids, suggesting inadequate initial symptom control and possible escalation toward treatments typically used for presumed inflammatory activity.^16^

Despite the central role of pain in ARDs, there are no comprehensive, evidence-based guidelines that address how analgesic medications should be sequenced, combined, or de-escalated across these conditions, particularly among patients with co-occurring COPCs. Most available data derive from single-disease cohorts or limited timeframes, offering an incomplete picture of real-world prescribing patterns across ARDs.^16,17^ As a result, clinical decision-making often occurs in the absence of contemporary, cross-disease evidence describing the extent of polypharmacy, variation across conditions, and the contribution of COPCs to medication burden. Rheumatologists routinely manage both inflammatory activity and chronic pain, making decisions about whether to escalate analgesics, switch classes, or layer additional therapies in the context of persistent symptoms and complex pain mechanisms.^18,19^ Yet without a clear understanding of current prescribing landscapes (e.g., how treatments are actually being sequenced and combined over time) it is difficult to determine where practice diverges, where risk accumulates, and where evidence-based guidance is most urgently needed. By systematically characterizing real-world pain treatment pathways, this work provides the empirical foundation necessary to inform the development of evidence-based recommendations and to support their practical implementation in routine rheumatologic care.

The primary objective of this study was to characterize real-world pain-related medication use following diagnosis of ARDs and to quantify the extent of analgesic polypharmacy in routine care. We further sought to determine whether medication burden differs across ARDs and among patients with COPCs, and to assess whether demographic factors such as age and sex are associated with higher levels of polypharmacy. Finally, we aimed to evaluate how prescribing patterns have evolved over time to contextualize current practice within broader historical trends. We hypothesized that (1) polypharmacy would be common in the year following diagnosis, (2) patients with COPCs would experience substantially greater medication burden than those without COPCs, and (3) prescribing complexity would vary meaningfully across ARDs.

## METHODS

### Data Source.

We used the Merative^™^ MarketScan^®^ Commercial Claims and Encounters Database, which includes longitudinal medical and outpatient pharmacy claims for commercially insured adults across the United States.^20,21^ The database captures enrollment information and detailed inpatient, outpatient, and pharmacy claims from a wide range of employers and health plans. All data are deidentified and HIPAA-compliant. Because this study relied on existing, deidentified data, it was exempt from institutional review board review.

### Study Population.

We identified adult patients in the outpatient setting using ICD-9-CM and ICD-10-CM diagnosis codes for ankylosing spondylitis, psoriatic arthritis, rheumatoid arthritis, Sjögren’s disease, systemic lupus erythematosus, or systemic sclerosis. Patients were eligible if they had at least one diagnosis code assigned by a specialist (rheumatologist, dermatologist, or nephrologist). Case definitions for each ARD are provided elsewhere.^17^ We excluded individuals without documented drug benefits, those lacking one year of continuous enrollment before the first diagnosis code, and those lacking one year of continuous enrollment after the first diagnosis code Because multiple overlapping ARD diagnoses were rare (< 2%), we retained only the first recorded ARD diagnosis for each patient.

### Outcome Measures.

We defined a list of pain medications across ten pharmacologically distinct categories: opioids, antidepressants, nonsteroidal anti-inflammatory drugs (NSAIDs), anticonvulsants, skeletal muscle relaxants, topical analgesics, acetaminophen, benzodiazepines, antimigraine agents, and cannabinoids.^17^ For each patient, we used pharmacy claims data to identify all prescriptions that were filled within one year of the first ARD diagnosis in any of these ten categories. For each prescription, we recorded only the pharmacologically active substance. Our analyses thus focus on unique active substances and do not differentiate between medications of different brand names that use the same active substance.

To define pain polypharmacy, we used five distinct approaches, each capturing a unique dimension of medication use. First, we calculated the total number of distinct pain medications (i.e. the number of distinct active substances) filled within one year after ARD diagnosis for each patient, providing a simple count-based measure of polypharmacy.^22^ Second, we defined minor polypharmacy as a patient having five or more distinct pain medications (≥ 5 medications) filled within one year of diagnosis. Third, we defined severe polypharmacy as a patient having ten or more distinct pain medications (≥ 10 medications) filled within one year of diagnosis. Fourth, to capture the breadth of medication classes prescribed independent of individual medication count, we used the ten broader categories of pain medications. We calculated how many of these distinct medication categories were used by each patient, ranging from 0 to 10 categories. Fifth, we applied the Medication Quantification Scale IV (MQS-IV),^23–26^ a validated instrument that quantifies cumulative medication detriment defined as the potential for adverse effects, including toxicity, drug-drug interactions, addiction, and tolerance. Each medication was assigned a detriment weight (i.e. a numeric value) based on established evidence: opioids (7.0), benzodiazepines (8.0), cannabinoids (6.0), NSAIDs (5.5), anticonvulsants (5.5), antidepressants (4.5), skeletal muscle relaxants (4.5), antimigraine agents (4.5), topical analgesics (2.0), and acetaminophen (2.0).^23,24^ Medications that have greater potential for harm are assigned a higher detriment weight. The total MQS for each patient is then calculated by summing all the detriment scores across all of that patient’s medications. Higher values thus represent increased medication use and greater potential for harm.^25,27^

### Variables of Interest.

We identified COPCs^6^ using validated ICD-9-CM and ICD-10-CM codes for fibromyalgia, irritable bowel syndrome, urologic chronic pelvic pain syndromes, vulvodynia (for females), chronic migraine, chronic tension-type headache, temporomandibular disorders, chronic low back pain, chronic fatigue syndrome, and endometriosis (for females). Demographic characteristics included age (examined continuously and categorized as ≤ 30, 31–40, 41–50, 51–60, and 61–64 years), sex, and geographic region (Northeast, North Central, South, West).

### Analysis Plan.

For each definition, we calculated either the frequency of polypharmacy or the mean level of polypharmacy. We calculated the mean MQS, the mean number of pain medications, the mean number of pain medication categories, the proportion of patients with at least five medications, and the proportion of patients with at least ten medications. We then examined how the severity of polypharmacy has changed over time. For each calendar year from 2008 through 2021, we calculated the mean MQS, the mean number of pain medications, the mean number of medication categories for each COPC, the proportion of patients with at least five medications, and the proportion of patients with at least ten medications. For each proportion or mean, we calculated a 95% confidence interval using a normal approximation. Finally, to assess whether polypharmacy changed following the 2015 national prescribing guideline update, we classified patients into two diagnosis-year groups: pre-2015 (diagnosed before 2015) and 2015 or later (diagnosed in 2015 or later). For each of five polypharmacy definitions, we estimated the period-specific outcome using year-equal weighting, such that each calendar year contributed equal total weight to the estimate. We then tested the difference in year-equally-weighted means (for continuous measures) or year-equally-weighted proportions (for binary measures) between the two periods. Standard errors incorporated within-year sample size and within-year variability (via the sampling variance of the annual mean or annual proportion), and two-sided p-values were computed using a Normal approximation. All analyses were conducted using R 4.1.1 and SAS 9.4 software.

## RESULTS

We identified 149,742 adults with newly diagnosed ARDs who met all inclusion criteria (Supplementary Fig. 1). The mean age of the cohort was 48.2 years (SD 10.9), and 75.7% were female (Table 1). Overall, 86,186 patients (57.6%) had at least one diagnosed COPC in the year before and after ARD diagnosis. Patients with COPCs were more likely to be female than those without COPCs (79.4% vs. 70.7%). Age distributions were broadly similar between groups. Geographic distribution was comparable across groups, with nearly half of patients residing in the Southern United States.

Pain-related polypharmacy was common across all ARDs, regardless of the metric used to define medication burden (Table 2). Across the full cohort, the mean MQS IV score in the year following diagnosis was 3.8 (SD 5.7). Patients filled a mean of 9.0 (SD 13.1) distinct pain medications and used an average of 2.1 (SD 1.9) pharmacologically distinct pain medication categories. Nearly half of all patients (47.9%) met criteria for minor polypharmacy, defined as filling ≥ 5 distinct pain medications within one year of diagnosis. Severe polypharmacy was also frequent, with 30.5% of patients filling ≥ 10 distinct pain medications during the same period.

Substantial heterogeneity in pain medication burden was observed across autoimmune rheumatic diseases (Table 2). Patients with ankylosing spondylitis exhibited the highest overall burden, with a mean MQS-IV score of 4.9 (SD 6.6), a mean of 11.6 (SD 15.3) distinct pain medications, and the greatest breadth of medication classes (mean 2.5 categories). In this group, 57.5% of patients met criteria for minor polypharmacy and 37.9% met criteria for severe polypharmacy. Patients with rheumatoid arthritis and systemic lupus erythematosus demonstrated similarly high medication burden. Approximately half of patients with rheumatoid arthritis (49.7%) and systemic lupus erythematosus (46.8%) filled ≥ 5 distinct pain medications, and nearly one-third met criteria for severe polypharmacy. Mean MQS-IV scores were 3.9 (SD 5.6) for rheumatoid arthritis and 4.0 (SD 6.0) for systemic lupus erythematosus. In contrast, patients with systemic sclerosis had the lowest medication burden across all measures, with a mean MQS-IV score of 2.9 (SD 4.9), a mean of 6.8 (SD 11.3) distinct pain medications, and only 23.3% meeting criteria for severe polypharmacy. Patients with psoriatic arthritis and Sjögren’s disease exhibited intermediate levels of medication use.

**Figure 1:**
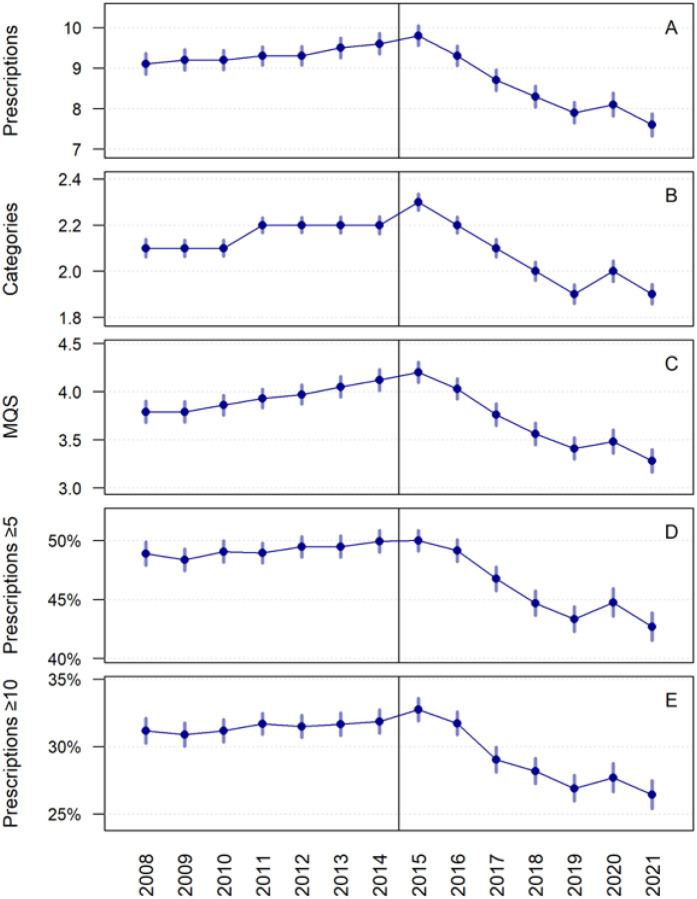
Polypharmacy trends across time. Means or percentages with 95% confidence intervals. A) Mean number of prescriptions; B) Mean number of distinct medication classes; C) Mean Medication Quantification Scale; D) Percent of Patients with Minor Polypharmacy; E) Percent of patients with major polypharmacy

Across all ARDs, pain medication burden increased steadily from 2008 through approximately 2014–2015, followed by a gradual decline thereafter (Fig. 1). This pattern was consistent across all polypharmacy metrics, including the mean number of distinct pain medications, the mean number of medication categories, mean MQS-IV scores, and the proportion of patients meeting criteria for minor and severe polypharmacy. When comparing the period prior to 2015 (2008–2014) with the period after 2015 (2015–2024), statistically significant reductions were observed across all measures (Supplementary Table). The mean number of distinct pain medications declined from 9.31 to 8.53 (difference − 0.79; p < 0.0001), and the mean number of medication categories decreased from 2.16 to 2.06 (difference − 0.10; p < 0.0001). Mean MQS-IV scores decreased from 3.93 to 3.67 (difference − 0.26; p < 0.0001). Similarly, the proportion of patients meeting criteria for minor polypharmacy declined from 49% to 46%, and the proportion meeting criteria for severe polypharmacy declined from 31% to 29% (both p < 0.0001). Despite these reductions, absolute levels of medication use remained high throughout the study period.

**Figure 2:**
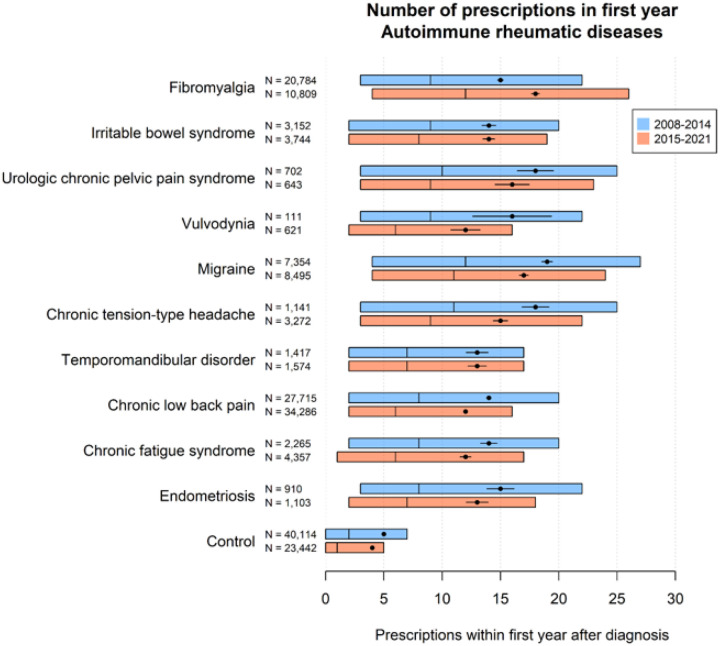
Number of prescriptions by COPC Red and blue bars show the first, second, and third quartiles. Black points show means with 95% confidence intervals.

Pain medication burden varied substantially by the presence of COPCs (Fig. 2). Patients with migraines, chronic tension type headache, urologic chronic pelvic pain syndrome, and fibromyalgia received a higher mean number of pain medications than patients with other COPCs. Patients with no COPC received far fewer pain medications than patients with COPCs. The mean and median number of medications decreased from pre-2015 to post-2015 within most COPC patients but increased among fibromyalgia patients. Disease-specific analyses demonstrated similar patterns (Supplementary Figs. 2–7). Among fibromyalgia patients, the mean number of medications increased after 2015 within all diseases except ankylosing spondylitis.

## DISCUSSION

To our knowledge, this is the first study to characterize pain related polypharmacy in ARD. We provide the most comprehensive evaluation to date of pain-related prescribing patterns, medication volume, and polypharmacy across multiple ARDs and chronic overlapping pain phenotypes using a large, national study of commercially insured adults with newly diagnosed ARDs. Using longitudinal data spanning 15 years and five complementary measures of medication burden, we found that pain-related polypharmacy remains common despite recent declines in overall prescribing intensity. Nearly half of patients filled ≥ 5 distinct pain medications within the first year of diagnosis, and almost one-third filled ≥ 10 distinct pain medications. Although all polypharmacy metrics declined modestly after 2015 (e.g., mean distinct pain medications from 9.31 to 8.53; all p < 0.0001), absolute levels remained high at 46–29% meeting minor/severe polypharmacy criteria. This decline occurred despite national claims data showing stable/increasing non-opioid analgesic prescribing^17,28^ (e.g., NSAIDs stable, gabapentinoids doubled 2009–2016 in MarketScan) amid opioid reductions, suggesting ARD-specific factors such as greater DMARD/bDMARD uptake, improved inflammatory control, or de-intensification of short-term peri-diagnostic analgesics (e.g., NSAIDs tapering post-DMARD initiation).

Our findings demonstrate that substantial pain medication exposure occurs within the first year following ARD diagnosis. However, very little is known about the contributions of analgesics to polypharmacy in rheumatology. In a recent cohort of 111 newly diagnosed patients (81 RA, 30 SLE), polypharmacy prevalence increased from 43% to 74% in RA and from 47% to 73% in SLE over two years, with mean medication counts rising from 4.6 to 6.9 in RA and from 6.5 to 7.8 in SLE, while overall medication adherence exceeded 85%.^29^ Analgesic use peaked at diagnosis, with mean NSAID use highest at baseline and declining over follow-up (RA: 0.58 at baseline vs 0.19 at two years; SLE: 0.27 vs 0.17), alongside a marked reduction in corticosteroid use in RA (0.22 to 0.04).^29^ This pattern suggests that early polypharmacy largely reflected short-term reliance on symptomatic pain management around diagnosis, which was subsequently tapered as disease-modifying therapy intensified.^29^ Consistent with this, the association between polypharmacy and improved disease control in RA disappeared when DMARDs were excluded, indicating that analgesics contributed to early medication burden but did not drive sustained improvements in disease activity.^29^ This study was limited to patients with RA and SLE and those that volunteered to be included in an inception cohort of newly diagnosed subjects; the authors interpreted these findings as consistent with more aggressive (more trial and error approaches) ‘treat-to-target’ therapy for RA rather than a “wait and see” approach which is more likely utilized in SLE. However, sequence-based analyses in SLE demonstrate ongoing cycling through pain therapies despite overall declines in prescribing intensity: among 769 patients, 48–61% of those initiated on acetaminophen transitioned through multiple pain medication classes within four treatment cycles, and nearly half (45.3%) continued to meet criteria for pain polypharmacy (≥ 5 pain prescriptions) in the more recent period.^16^ These patterns suggest that while early polypharmacy may partly reflect transient analgesic use at diagnosis, persistent medication cycling in rheumatology reflects unresolved pain that necessitates repeated therapeutic escalation, sustaining medication burden even as high-risk agents such as opioids and steroids are increasingly avoided.

The presence and type of COPC strongly shaped prescribing patterns over time, with fibromyalgia demonstrating a distinct and concerning divergence characterized by increasing polypharmacy relative to other pain conditions. Across all ARDs, patients with fibromyalgia consistently had the highest medication burden, including a greater mean number of distinct pain medications, higher MQS-IV scores, and broader use of pain medication classes compared with patients with other COPCs. Notably, within ankylosing spondylitis, rheumatoid arthritis, and systemic lupus erythematosus, fibromyalgia was associated with a steeper rise in pain medication use after 2015, diverging from the overall post-2015 decline observed in the broader ARD population. In general, patients with fibromyalgia receive more medications overall and for longer durations than those with similar comorbidity profiles but without fibromyalgia, including higher use of opioids (11–26% chronic/long-term exposure), nervous system agents, and psychotropics. For example, in a cross-sectional study of 159 patients, the fibromyalgia group (n = 59) showed higher total drug counts, longer treatment durations, more nervous system drugs, exclusive use of major opioids (13.6%), high benzodiazepine use (74%) alongside opioids, and greater psychiatric care involvement (45.8% vs. 3–15.6% in comparator groups).^30^ Among 28,552 fibromyalgia patients newly initiating opioids, 26% (7,369 patients) progressed to long-term opioid therapy within 1 year; similarly, 11.3% of 245,758 U.S. fibromyalgia patients received chronic opioid therapy.^31,32^ These observations also raise the possibility of diagnostic–treatment circularity. Patients presenting with complex, refractory pain and higher medication burden may be more likely to receive a fibromyalgia diagnosis, and the diagnostic label itself may subsequently influence prescribing behavior. Thus, the association between fibromyalgia and escalating polypharmacy may reflect both underlying nociplastic vulnerability and prescribing responses to perceived treatment resistance. Disentangling these mechanisms is critical, particularly in the early period following ARD diagnosis when treatment trajectories are being established and medication burden rapidly accumulates. These patterns highlight fibromyalgia as a high-risk phenotype for adverse drug events and underscore the need for longitudinal studies that can distinguish predisposing pain vulnerability from medication-driven amplification of risk.

Ankylosing spondylitis represents a distinct therapeutic context among autoimmune rheumatic diseases. Unlike RA or SLE, where NSAIDS are typically used for symptom relief, NSAIDs are recommended as first-line and disease-specific therapy in ankylosing spondylitis and may be continued as part of long-term management.^33–35^ Therefore, stepwise escalation through multiple NSAIDs is common in early treatment, particularly within the first 12 months after diagnosis. This treatment structure may partially explain the higher levels of polypharmacy observed in spondyloarthritis populations. At the same time, prescribing patterns in ankylosing spondylitis may also reflect the central role of chronic inflammatory back pain, which differs from the predominant symptom profiles of RA and SLE.^36^ Persistent axial pain may lead to additional use of muscle relaxants, neuropathic agents, opioids, or other adjunctive medications alongside disease-modifying therapy.^37^ Therefore, the elevated medication burden in this population may arise from both guideline-driven NSAID use and the complexity of managing chronic back pain. Clarifying whether polypharmacy risk is primarily driven by treatment algorithms, back pain–related symptom burden, or coexisting pain vulnerability will require further study, particularly during the early period after diagnosis when medication trajectories are being established.

Emerging evidence from RA cohorts suggests that polypharmacy functions less as an isolated prescribing problem and more as a surrogate marker of disease complexity and comorbidity burden, with important implications for long-term outcomes. A few studies suggest that over the course of time, RA patients attempt to withdraw medications (either on their own, or with physician consent) because of fear of long-term consequences.^38,39^ How this affects polypharmacy behavior is not known. In an urban UK early rheumatoid arthritis cohort (n = 497), patients with polypharmacy (≥ 2 non-RA medications) achieved DAS28 remission at 1 year in 32.1% of cases versus 67.9% without polypharmacy (P = 0.07), and 45.0% versus 56.3% at 5 years (P = 0.03).^40^ Polypharmacy conferred 40% lower adjusted odds of remission at 5 years (OR 0.60, 95% CI 0.38–0.94; P = 0.03) and 43% lower at 10 years (OR 0.57, 95% CI 0.34–0.94; P = 0.02).^40^ With serious adverse event incidence at 61 per 1,000 person-years and 86.4% of cases among those with polypharmacy versus 49.8% without (P = 0.03), these data affirm polypharmacy as a comorbidity surrogate linked to inferior treatment response for our MarketScan ARD analyses.^40^ In contrast, in a cohort of 1,101 rheumatoid arthritis patients, polypharmacy was prevalent (11%) and strongly associated with older age, longer disease duration, higher DAS28, and greater HAQ scores.^40^ Patients with polypharmacy faced a markedly elevated risk of unplanned hospitalization (adjusted HR 3.1, 95% CI 2.1–4.5), with infections as the leading cause (29%), while corticosteroid use independently doubled admission risk (HR 1.7, 95% CI 1.2–2.4).^40^ Although adverse drug reactions contributed to only 7% of admissions (few directly avoidable), these findings position polypharmacy as a marker of comorbidity burden rather than primary toxicity driver, underscoring its value for risk stratification in ARD polypharmacy studies.^40^ These findings also support interpreting the high prevalence of pain-related polypharmacy observed in our MarketScan ARD cohort as a clinically meaningful risk signal for patient management, one that captures disease severity and multimorbidity rather than medication toxicity alone, and that may help identify patients at heightened risk for poor outcomes and increased healthcare utilization.

This study has limitations inherent to the use of administrative claims data. First, while claims data provide accurate records of filled prescriptions, they do not verify medication adherence. It is possible that some patients did not consume all medications dispensed, potentially leading to an overestimation of actual physiological exposure. Conversely, our analysis did not capture over-the-counter medications (such as NSAIDs or acetaminophen purchased without a prescription) or illicit substances, which may lead to an underestimation of the total analgesic burden. Second, our reliance on ICD-9-CM and ICD-10-CM codes for case identification is subject to misclassification bias. Although we required diagnosis codes from specialists (rheumatologists, dermatologists, or nephrologists) and used validated algorithms to minimize error, the severity of ARD disease activity and the specific severity of pain symptoms (e.g., pain intensity scores) are not available in administrative claims. Consequently, we could not adjust for disease severity or pain intensity, making it difficult to determine whether higher polypharmacy rates were driven by refractory inflammatory disease or central sensitization. Third, because the MarketScan database covers a commercially insured population, our findings may not be generalizable to patients with public insurance (e.g., Medicare, Medicaid) or the uninsured. These populations often have different demographic profiles, comorbidity burdens, and access to care, which could influence prescribing patterns. Finally, while we identified COPCs using diagnosis codes, conditions like fibromyalgia are often underdiagnosed in routine practice; thus, the influence of COPCs on polypharmacy may be even greater than reported here.

This study has several strengths. First, the large sample size allowed us to examine prescribing patterns across multiple distinct ARDs and specific COPCs with a high degree of statistical power, overcoming the limitations of previous single-disease or single-center cohorts.^16^ Second, our methodological approach captures the complexity of pain management more effectively than simple prescription counts. We employed five complementary metrics, including the validated MQS-IV to quantify not just the volume of medications, but also the pharmacological breadth and cumulative potential for adverse effects. This multidimensional assessment provides a more clinically relevant picture of medication burden than previous studies. Third, the longitudinal nature of our data, spanning from 2008 through 2022, allowed us to contextualize current prescribing practices within a broader historical framework. This enabled us to identify significant shifts in practice, specifically the rise in medication burden through 2014–2015 and the subsequent decline, likely reflecting national trends in opioid stewardship. Finally, by specifically stratifying outcomes by the presence of individual COPCs, we highlighted the distinct and escalating trajectory of polypharmacy in patients with fibromyalgia, offering actionable insights for targeted deprescribing and risk stratification.

In summary, the persistence of high pain-related polypharmacy in ARDs, even amid overall declines in prescribing intensity, likely reflects the fundamental challenge of managing pain that is mechanistically heterogeneous and often decoupled from inflammatory disease activity.^41^ For many patients, particularly those with co-occurring COPCs, pain is driven by centralized or mixed mechanisms that do not reliably respond to anti-inflammatory or peripherally targeted analgesic strategies.^15^ In this context, clinicians are often left with few evidence-based options beyond sequential or additive pharmacotherapy, resulting in trial-and-error escalation across multiple medication classes.^16^ Importantly, post-2015 reductions in opioid and glucocorticoid use do not appear to have translated into proportional reductions in overall medication burden; instead, these shifts may have altered the composition of pain regimens rather than their cumulative detriment.^17^ Our findings suggest that polypharmacy in ARDs is not simply a legacy of high-risk prescribing practices, but a structural consequence of attempting to manage persistent, multifactorial pain within disease-centered care models that lack robust guidance for pain phenotyping and mechanism-informed treatment selection. In fact, major clinical guidelines for ARDs, such as NICE recommendations for RA prioritize DMARDs with minimal guidance on chronic pain management, limited to short-term NSAIDs, hand exercises, and specialist physiotherapy access.^13,42^ EULAR guidelines emphasize inflammatory control but offer sparse, nonspecific pain directives, often deferring to multimodal nonpharmacologic approaches without detailed chronic pain protocols.^42^ The most recent ACR guidelines on pain management were published almost sixteen years ago.^43^ These gaps highlight the need for updated rheumatology pain guidelines, as current ones inadequately address persistent nociplastic or centralized pain.^19^ These efforts are essential so that patients avoid accruing substantial medication exposure despite adherence to contemporary prescribing norms, and underscore the need to rethink how chronic pain is conceptualized and treated alongside autoimmune disease.

## Supplementary Material

Supplementary Files

This is a list of supplementary files associated with this preprint. Click to download.

- SupplementaryFiguresandTables.docx

## Figures and Tables

**Table 1:** Sociodemographic Characteristics among ARD patients

N (%)	Total	ARD with chronic pain conditions	ARD without chronic pain conditions
	149,742 (100)	86,186 (57.6)	63,556 (42.4)
Age, mean (SD)	48.2 (10.9)	48.0 (10.8)	48.4 (11.1)
≤ 30 years	11,840 (7.9)	6,609 (7.7)	5,231 (8.2)
31–40 years	22,627 (15.1)	13,482 (15.6)	9,145 (14.4)
41–50 years	41,296 (27.6)	24,567 (28.5)	16,729 (26.3)
51–60 years	57,923 (38.7)	32,873 (38.1)	25,050 (39.4)
61–64 years	16,056 (10.7)	8,655 (10.0)	7,401 (11.6)
Female (%)	113,396 (75.7)	68,465 (79.4)	44,931 (70.7)
Enrollment Regions			
Northeast	27,761 (18.5)	15,520 (18.0)	12,241 (19.3)
North CentralMidwest	27,752 (18.5)	15,688 (18.2)	12,064 (19.0)
South	71,138 (47.5)	41,893 (48.6)	29,245 (46.0)
West	21,675 (14.5)	12,283 (14.3)	9,392 (14.8)
Unknown	1,416 (0.9)	802 (0.9)	614 (1.0)

**Table 2:** Polypharmacy Status among ARD patients

N (%)	Total	AS	PsA	RA	SLE	SjD	SSc
	149,742 (100)	11,410 (7.6)	23,182 (15.5)	74,918 (50.0)	17,735 (11.8)	18,858 (12.6)	3,639 (2.4)
Polypharmacydefinitions							
MQS-IV (Mean, SD)	3.8 (5.7)	4.9 (6.6)	3.4 (5.2)	3.9 (5.6)	4.0 (6.0)	3.5 (5.6)	2.9 (4.9)
Number of distinctprescriptions (Mean,SD)	9.0 (13.1)	11.6 (15.3)	8.0 (12.0)	9.2 (13.0)	9.3 (13.9)	8.3 (13.0)	6.8 (11.3)
% ≥5 medications	71,801 (47.9)	6,560 (57.5)	10,339 (44.6)	37,221 (49.7)	8,298 (46.8)	8,019 (42.5)	1,364 (37.5)
% ≥10 medications	45,701 (30.5)	4,323 (37.9)	6,321 (27.3)	23,546 (31.4)	5,526 (31.2)	5,136 (27.2)	849 (23.3)
Number of distinctmedicationcategories (Mean,SD)	2.1 (1.9)	2.5 (2.0)	1.9 (1.8)	2.2 (1.9)	2.2 (2.0)	2.0 (1.9)	1.7 (1.8)

* AS: Ankylosing spondylitis; PsA: Psoriatic arthritis; RA: Rheumatoid arthritis; SLE: Systemic lupus erythematosus; SjD: Sjögren's disease; SSC: Systemic sclerosis.
